# The combination of ribose and adenine promotes adenosine release and attenuates the intensity and frequency of epileptiform activity in hippocampal slices: Evidence for the rapid depletion of cellular ATP during electrographic seizures

**DOI:** 10.1111/jnc.14543

**Published:** 2018-09-10

**Authors:** Jessicka Hall, Bruno G. Frenguelli

**Affiliations:** ^1^ School of Life Sciences The University of Warwick Coventry UK

**Keywords:** Adenosine, ATP, epilepsy, purines, RibAde, seizures

## Abstract

In addition to being the universal cellular energy source, ATP is the primary reservoir for the neuromodulator adenosine. Consequently, adenosine is produced during ATP‐depleting conditions, such as epileptic seizures, during which adenosine acts as an anticonvulsant to terminate seizure activity and raise the threshold for subsequent seizures. These actions protect neurones from excessive ionic fluxes and hence preserve the remaining cellular content of ATP. We have investigated the consequences of manipulation of intracellular ATP levels on adenosine release and epileptiform activity in hippocampal slices by pre‐incubating slices (3 h) with creatine (1 mM) and the combination of ribose (1 mM) and adenine (50 μM; RibAde). Creatine buffers and protects the concentration of cellular ATP, whereas RibAde restores the reduced cellular ATP in brain slices to near physiological levels. Using electrophysiological recordings and microelectrode biosensors for adenosine, we find that, while having no effect on basal synaptic transmission or paired‐pulse facilitation, pre‐incubation with creatine reduced adenosine release during Mg^2+−^free/4‐aminopyridine‐induced electrographic seizure activity, whereas RibAde increased adenosine release. This increased release of adenosine was associated with an attenuation of both the intensity and frequency of seizure activity. Given the depletion of ATP after injury to the brain, the propensity for seizures after trauma and the risk of epileptogenesis, therapeutic strategies elevating the cellular reservoir of adenosine may have value in the traumatized brain. Ribose and adenine are both in use in man and thus their combination merits consideration as a potential therapeutic for the acutely injured central nervous system.

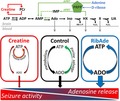

**Abbreviations used**
4‐AP: 4‐aminopyridine
5‐HT: 5‐hydroxytryptamine/serotonin
8‐CPT/CPT: 8‐cyclopentyltheophylline
aCSF: artificial cerebrospinal fluid
Ado: adenosine
ADP: adenosine diphosphate
AMP: adenosine monophosphate
ANOVA: one‐way analysis of variance
APRT: adenine phosphoribosyl‐transferase
ATP: adenosine triphosphate
fEPSP: field excitatory post‐synaptic potential
H_2_O_2_: hydrogen peroxide
HPRT: hypoxanthine phosphoribosyl‐transferase
Hx: hypoxanthine
Ino: inosine
K^+^: potassium
Mg^2+^: magnesium
n: number of observations
ns: not significant
OGD: oxygen/glucose deprivation
pA: picoamp
p: probability
PRPP: 5‐phosphoribosyl‐1‐pyrophosphate
Pt/Ir: platinum/iridium
Rib‐1‐P: ribose‐1‐phosphate
RibAde: ribose and adenine
SEM: standard error of the mean
UA: Uric acid
X: xanthine

The energy budget of the brain is spent largely on the re‐establishment of ionic homeostasis associated with synaptic transmission and neuronal signalling (Harris *et al*. 2012). The energy currency occurs in the form of adenosine triphosphate (ATP), which, after its sequential dephosphorylation, gives rise to the neuromodulator adenosine (Fig. 1). Adenosine reduces neuronal energy demand via the adenosine A_1_ receptor‐mediated inhibition of glutamatergic excitatory synaptic transmission and hyperpolarization of post‐synaptic neurones. (Dunwiddie and Masino 2001; Sebastiao and Ribeiro 2015). The accumulation of extracellular adenosine thus reflects the situation where energy demand has outstripped energy production and where, as a neuroprotective strategy, energy demands associated with neuronal activity must be minimized until as such time as ATP production resumed and/or ATP levels restored.

**Figure 1:**
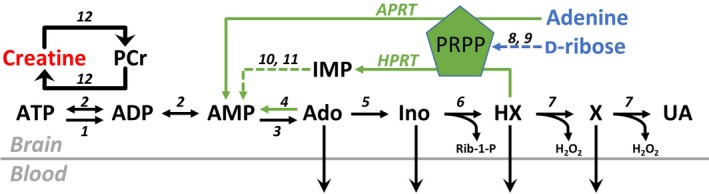
ATP metabolism and synthesis via the cytosolic purine salvage pathway. Under conditions of energy depletion, ATP is metabolized to adenosine (Ado), inosine (Ino), hypoxanthine (HX) and xanthine (X), which can, via equilibrative transporters, leave cells and enter the bloodstream. Direct cellular release of ATP and subsequent extracellular metabolism by ectonucleotidases (not shown) provides another source of extracellular adenosine and purine loss to the bloodstream. Purine salvage (green arrows) restores adenine nucleotide levels via adenine phosphoribosyl‐transferase (APRT, EC 2.4.2.7; adenine to AMP) and hypoxanthine phosphoribosyl‐transferase (HPRT, EC 2.4.2.8; hypoxanthine to inosine monophosphate, IMP). This reaction requires 5‐phosphoribosyl‐1‐pyrophosphate (PRPP), a product of the pentose phosphate pathway that gives rise to ribose‐5‐phosphate, which can also arise from the isomerization of inosine‐derived ribose‐1‐phosphate (Rib‐1‐P) by phosphopentomutase (EC 5.4.2.7), and the action of ribokinase (EC 2.7.1.15) on D‐ribose. Creatine can be converted to phosphocreatine (PCr), which acts as a phosphate donor to ADP to regenerate ATP, thus buffering ATP levels and preventing accumulation of ATP metabolites. 1, ATPases; 2, adenylate kinase (EC 2.7.4.3); 3, cytosolic 5′nucleotidase (EC 3.1.3.5); 4, adenosine kinase (EC 2.7.1.20); 5, adenosine deaminase (EC 3.5.4.4); 6, purine nucleoside phosphorylase (EC 2.4.2.1); 7, xanthine oxidase (EC 1.17.3.2); 8, ribokinase (EC 2.7.1.15); 9, phosphoribosylpyrophosphate synthetase (EC 2.7.6.1); 10, adenylosuccinate synthetase (EC 6.3.4.4); 11, adenylosuccinate lyase (EC 4.3.2.2); 12, creatine kinase (EC 2.7.3.2). H_2_O_2_, hydrogen peroxide; UA, uric acid. Colour coding for creatine (red) and D‐ribose/adenine (blue) are used throughout the data figures.

Such situations commonly arise in acute pathological conditions, for example cerebral ischaemia where ATP depletion and the release of adenosine occurs very rapidly after the onset of occlusion of a blood vessel *in vivo*, or the removal of oxygen and glucose from the perfusing medium *in vitro* (Dale and Frenguelli 2009; Pedata *et al*. 2016). During seizure activity the extracellular accumulation of adenosine (During and Spencer 1992; Dunwiddie 1999; Dale and Frenguelli 2009) likely results from rapid, potentially very local (Wall and Richardson 2015; Frenguelli and Wall 2016), intracellular depletion of ATP, as well as the extracellular metabolism of ATP released in an activity‐dependent manner (Dale and Frenguelli 2009). Under both these conditions, the activation of adenosine A_1_ receptors suppresses neuronal activity in an attempt to minimize cellular energy demand. The intersection of multiple ATP‐depleting and adenosine‐releasing insults occurs with the prevalence of seizures after both stroke (Chung 2014) and traumatic brain injury (Rao and Parko 2015).

Such insults cost the brain dearly in terms of repeated demands on the energy currency. This situation is exacerbated by the loss from the brain into the bloodstream of adenosine and subsequent metabolites (Weigand *et al*. 1999; Tian *et al*. 2017). One such metabolite is hypoxanthine, which is utilized by one branch of the purine salvage pathway (Ipata *et al*. 2011), the primary route by which adenine nucleotides are synthesized in the brain (Fig. 1). The loss of ATP metabolites from the brain thus robs the brain of the substrates with which to resynthesize ATP and likely explains both the prolonged depletion of cerebral ATP levels after injury, and the greater impact of secondary insults, such as spreading depolarization, on survival and prognosis (Kirino 2002; Strong *et al*. 2002; Pearson *et al*. 2003; Toth *et al*. 2016; Dreier *et al*. 2017; Frenguelli 2017).

We have previously shown that the provision of both the sugar backbone and purine moiety of ATP (ribose and adenine respectively; ‘RibAde’) restored the reduced ATP levels in acutely prepared brain slices to values found *in vivo* (zur Nedden *et al*. 2011). This likely occurred either via the action of the purine salvage enzyme adenine phosphoribosyl‐transferase (EC 2.4.2.7) and the direct formation of AMP, or via deamination of adenine to hypoxanthine and the action of hypoxanthine phosphoribosyl‐transferase (HPRT, EC 2.4.2.8). We have further shown that this elevated reservoir of ATP translates into greater release of adenosine in response to electrical stimulation of afferent fibres (zur Nedden *et al*. 2011), and oxygen/glucose deprivation (OGD) in hippocampal slices (zur Nedden *et al*. 2014). The enhanced adenosine release, via the activation of adenosine A_1_ receptors, raised the threshold for the induction of long‐term potentiation (zur Nedden *et al*. 2011), and both hastened and prolonged the effects of brief OGD on excitatory synaptic transmission (zur Nedden *et al*. 2014). In contrast, buffering the breakdown of ATP with creatine reduced both adenosine release and the depressant effects of OGD on synaptic transmission, but delayed the anoxic depolarization and protected synaptic transmission from prolonged OGD (zur Nedden *et al*. 2014).

In this study, we explored the release of adenosine during *in vitro* seizure activity when the availability of intracellular ATP is experimentally influenced by both creatine, which buffers ATP decline, and RibAde, which elevates cellular ATP levels. We find that creatine resulted in reduced extracellular adenosine release during epileptiform activity, whereas RibAde increased the release of adenosine under these conditions. These manipulations had consequences for seizure activity in enhancing and reducing seizure activity respectively. These findings demonstrate that the source of extracellular adenosine during seizure activity likely arises, at least in part, from the metabolic pool of ATP, that this pool is reduced even during brief seizure activity, and that the pool is amenable to manipulation to influence neuronal activity. Moreover, these observations have implications for the traumatized brain, in, for example the provision of RibAde in the aftermath of injury to mitigate both the severity of post‐injury seizure activity and entry into an epileptogenic cascade.

## Methods

### Drugs and chemicals

Creatine (C0780), D‐ribose (R9629), adenine (A2786), adenosine (A9251), 8‐cyclopentyltheophylline (8‐CPT; C102), 5‐hydroxytryptamine hydrochloride (5‐HT; H9523) and 4‐aminopyridine (4‐AP; 275875) were purchased from Sigma Aldrich. 8‐CPT was dissolved in 0.1 M NaOH. Creatine, ribose and adenine were dissolved directly into artificial cerebrospinal fluid (aCSF). All salts for the aCSF were purchased from Fisher Scientific.

### Preparation of hippocampal slices

Seventy‐four 17‐ to 23‐day‐old male Sprague–Dawley rats, obtained form an in‐house colony, were used. To minimize suffering, rats were swiftly and humanely killed by cervical dislocation by a skilled and competent researcher. Animal procedures were in accordance with Schedule 1 of the UK Government Animals (Scientific Procedures) Act 1986 and were performed with approval from the University of Warwick Animal Welfare and Ethical Review Board (AWERB.30/13‐14). Animals were then decapitated and the brain was quickly removed and placed in ice‐cold aCSF containing (mM): NaCl (124), KCl (3), CaCl_2_ (2), NaHCO_3_ (26), NaH_2_PO_3_ (1.23), D‐glucose (10) and MgSO_4_ (1) with an additional MgCl_2_ (10); pH 7.4. Parasagittal hippocampal brain slices (400 μm) were cut on a vibratome (Microm HM 650 V microtome) (zur Nedden *et al*. 2011; zur Nedden *et al*. 2014) and placed in 100 or 250 mL incubation chambers in which aCSF circulated around the slices. Slices were kept at 34°C and bubbled with 95% O_2_/5% CO_2_ for at least 3 h before use (Edwards *et al*. 1989). Slices from a given animal were incubated in either standard 1 mM Mg^2+^‐containing aCSF, or in a standard aCSF containing either 1 mM creatine, or 1 mM D‐ribose plus 50 μM adenine (RibAde). While no formal randomization in assigning slices to experimental treatments was conducted, efforts were made to ensure that no systematic bias was introduced by, for example assigning a particular hemisphere or hippocampal location (dorsal, medial, ventral) to one experimental condition. Experiments were not conducted blind to the experimental treatment, and the study was not pre‐registered.

### Electrophysiological recordings and drug application

Post recovery, slices were submerged in a recording chamber, secured with a platinum harp with nylon threads and were perfused with oxygenated aCSF at a rate of ~6.0 mL/min and maintained at 32–33°C. To ensure adequate perfusion of the slice, the slice was placed upon a mesh platform that allowed aCSF to flow below and above the slice (Etherington and Frenguelli 2004). A twisted bipolar Teflon‐coated tungsten wire electrode (100 μm diameter) was placed in stratum radiatum in area CA1. Extracellular field excitatory post‐synaptic potentials (fEPSPs) were recorded with a glass microelectrode filled with aCSF. Slices were stimulated prior to the introduction of pro‐convulsant aCSF to ensure slice viability and stability of the recording, and to measure the effects of drugs on basal synaptic transmission. Stimulus parameters and acquisition and analysis of fEPSPs were under control of WinLTP software (WinLTP, RRID:SCR_008590; (Anderson and Collingridge 2007)). Epileptiform activity was captured and analysed using Spike2 software (Spike2 Software, RRID:SCR_000903; CED, Cambridge, UK) running simultaneously with WinLTP on the same PC. All pharmacological agents were bath‐applied.

### Biosensor recordings

Adenosine biosensors were used in these experiments to measure the release of purines in real‐time. Null biosensors, lacking the three enzymes necessary to metabolize adenosine to hydrogen peroxide, were used to detect the presence of electroactive interferents that could have contaminated the signal on the adenosine sensor. The signal from the null was subtracted from that of the adenosine sensor.

Adenosine (SBS‐ADO‐05‐50) and null (SBS‐NUL‐05‐50) biosensors (Pt/Ir wire of 50 μm in diameter and 500 μm in length) were purchased from Sarissa Biomedical Ltd (Coventry, UK) and were inserted into the slice in stratum radiatum of area CA1. The adenosine biosensors encapsulate a specific enzymatic cascade that metabolizes adenosine to produce hydrogen peroxide. The enzymes (adenosine deaminase, EC 3.5.4.4; purine nucleoside phosphorylase, EC 2.4.2.1 and xanthine oxidase, EC 1.17.3.2) are deposited on the screening layer where they are entrapped in a polymer matrix as described previously (Llaudet *et al*. 2003; Frenguelli and Wall 2016). Because of the nature of the enzymatic cascade present in the adenosine biosensors they are also capable of detecting not only adenosine but also its metabolites inosine, hypoxanthine and xanthine. Null sensors contain no enzymes and measure only non‐specific electroactive signals. After each experiment, sensors were withdrawn from slices and calibrated with 10 μM adenosine. The values from adenosine biosensors are given as micromolar prime (μM′) to reflect that the adenosine signal is a composite signal of adenosine and its metabolites (Frenguelli *et al*. 2007). Because of the highly localized nature of signals associated with epileptiform activity in brain tissue, it is difficult to consistently perform differential recordings between an adenosine sensor and an inosine sensor to yield a net adenosine signal (Wall and Richardson 2015; Frenguelli and Wall 2016). However, when such differential measurements between adenosine and inosine biosensors have been made they have demonstrated that the majority of the signal detected on the adenosine sensor during seizure activity was indeed adenosine (Etherington *et al*. 2009). The release of adenosine was measured over a time period of 15 min in nominally Mg^2+^‐free aCSF, after the appearance of three bursts or 10 min in 4‐AP, and after 10 min in 8‐CPT. Adenosine release associated with each burst was measured over the duration of the burst, that is from the start of the burst to its end; this likely underestimated the amount of release per burst as release often continued after the end of the burst. Release is given as the integral over these period of measurements in units of μM′s.

To confirm the patency of the biosensor electroactive interferent screening layer, biosensors were perfused with 10 μM 5‐HT. Biosensor measurements were only accepted and further processed if the biosensor current to 5‐HT did not exceed 150 pA. The current response of the simultaneously recorded null sensors was subtracted from the adenosine signal to reveal a net purine signal, the majority of which reflects the appearance of adenosine in the extracellular space (Etherington *et al*. 2009) and is therefore referred to as adenosine.

### Data analyses

Traces of extracellular epileptiform seizure activity were rectified, and the following measurements made: burst duration; inter‐burst interval, and, within a burst, the inter‐spike interval with the threshold inter‐spike interval being set to 50 ms to avoid double‐counting of rectified spikes. Bursting seizure activity was defined as periods of electrophysiological activity with intervening periods of quiescence – the inter‐burst interval. Bursting activity was deemed to have stopped when the inter‐spike interval became > 1.5 s. Basal synaptic transmission measurements were made, which included: fEPSP slope measurements during stimulus input/output curves at 50 μA increments from 50 to 300 μA; the pre‐synaptic fibre‐volley amplitude at the highest stimulus strength of 300 μA; the ratio of fEPSP slopes for paired stimulus pulses given 50 ms apart, and the change in fEPSP slope associated with the removal of Mg^2+^ from the aCSF. The integral of adenosine release was measured for null‐subtracted biosensor data and the values expressed as μM′s.

### Statistical analysis

Values are expressed as mean ± SEM. N values represent number of slices per condition, which in almost all cases is equivalent to the number of animals per condition. No sample size calculations were conducted. If more than two groups were to be assessed, a one‐way anova was used. For comparison of fEPSP slope during input/output curves, a two‐way mixed‐design anova, with treatment as the between‐group factor and stimulus strength as the repeated‐measures factor, was applied. Where significant differences were found across the groups, post hoc Bonferroni comparisons were made between groups. Graphs were drawn and statistical analyses were performed in OriginPro 2016 software (OriginPro, RRID:SCR_015636). Statistical significance was taken as *p* < 0.05.

## Results

### Influence of creatine and RibAde on basal synaptic transmission

**Figure 2:**
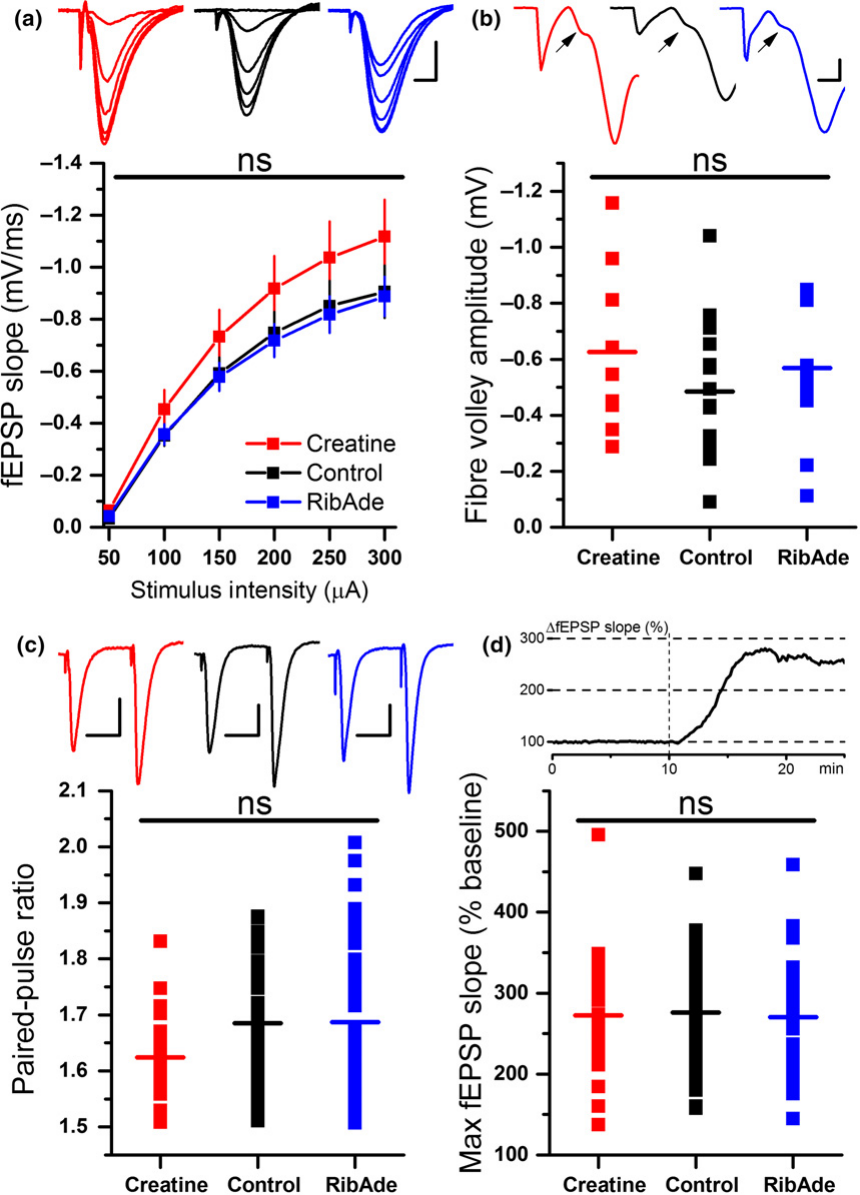
Influence of RibAde and creatine on excitatory synaptic transmission. (a) Input‐output curves of field excitatory post‐synaptic potential (fEPSP) slope versus stimulus strength (mean ± SEM) for control (black line and symbols; *n* = 56 slices) and creatine‐ (red; *n* = 28 slices) and RibAde‐treated slices (blue; *n* = 47 slices). Inset are representative fEPSPs at increasing stimulus strengths from each of the three conditions and colour‐coded as per the graph. There was no main effect of treatment on the input‐output curves (ns; *p* = 0.275) Scale bars measure 0.5 mV and 5 ms. (b) No significant difference (ns; *p* = 0.400) was observed in the pre‐synaptic fibre volley (measured at 300 μA stimulus strength; data from 9 to 17 slices) across the three conditions. The graph plots individual fibre volley amplitude for each experiment and condition with the mean for each depicted as a horizontal bar. Inset traces show representative fibre volleys indicated by an arrow and colour‐coded as per the graph. fEPSPs have been truncated at 5 ms after the onset of electrical stimulation (first downward deflection for each trace) and show the positive‐going population spike occasionally evoked at high (300 μA) stimulus strengths. Scale bar measures 0.5 mV and 1 ms. (c) Paired‐pulse facilitation was not influenced by RibAde or creatine (*p* = 0.088). The graph plots individual paired‐pulse ratios (50 ms inter‐pulse interval; *n* = 22–47 slices) for each experiment and condition with the mean for each depicted as a horizontal bar. Inset is the representative fEPSPs, evoked at 50 ms intervals, colour‐coded as per the graph. Scale bars measure 0.5 mV and 25 ms. (d) The enhancement of synaptic transmission caused by removal of Mg^2+^ from the artificial cerebrospinal fluid (aCSF) was not different in creatine‐ or RibAde‐treated slices compared to control slices (*p* = 0.927). The graph plots the maximal enhancement of the fEPSP after 15 min exposure to nominally Mg^2+^‐free aCSF (*n* = 19–51 slices) for each experiment and condition, with the mean for each depicted as a horizontal bar. Inset is a representative experiment showing the enhancement of the fEPSP (as a percentage of baseline) after removal of Mg^2+^ from the aCSF, which occurred at *t* = 10 min (broken vertical line).

Hippocampal slices were pre‐incubated for at least 3 h in standard aCSF, or aCSF supplemented with either creatine (1 mM) or RibAde (1 mM ribose and 50 μM adenine). Thereafter, slices were placed in a recording chamber and perfused with standard aCSF. Despite there being a trend for creatine‐treated slices to show stronger synaptic transmission, there was no significant main effect of treatment on the stimulus input/output curves (Fig. 2a; two‐way mixed‐design anova (treatment and stimulus strength) *F*_2,127_ = 1.202; *p* = 0.275). Treatment did not affect either the size of the pre‐synaptic fibre volley (Fig. 2b; measured at 300 μA stimulus strength; one‐way anova;* F*_2,33_ = 0.942; *p* = 0.400), or the paired‐pulse facilitation ratio measured at an inter‐pulse interval of 50 ms (Fig. 2c; one‐way anova;* F*_2,107_ = 2.49; *p* = 0.088). These data suggest that neither creatine nor RibAde affect pre‐synaptic function (axonal excitability and the probability of glutamate release respectively) or the post‐synaptic response to glutamate and are consistent with our previous observations (zur Nedden *et al*. 2011; zur Nedden *et al*. 2014).

After a period of baseline recording of the electrically evoked fEPSP, the aCSF was changed to one to which no Mg^2+^ was added (Mg^2+^‐free aCSF) in order to alleviate the Mg^2+^ block of the NMDA receptor and to increase tissue excitability (Etherington and Frenguelli 2004; Etherington *et al*. 2009; Lopatar *et al*. 2011). This manipulation caused an enhancement in the slope of the fEPSP, which was similar across the various treatments (Fig. 2d; creatine = 272.7 ± 18.4%, *n* = 19; control = 276.2 ± 9.0%, *n* = 51; RibAde = 270.3 ± 11.9%, *n* = 32; *F*_2,99_ = 0.076; *p* = 0.927).

### Bidirectional modulation of adenosine release during epileptiform activity by creatine and RibAde

**Figure 3:**
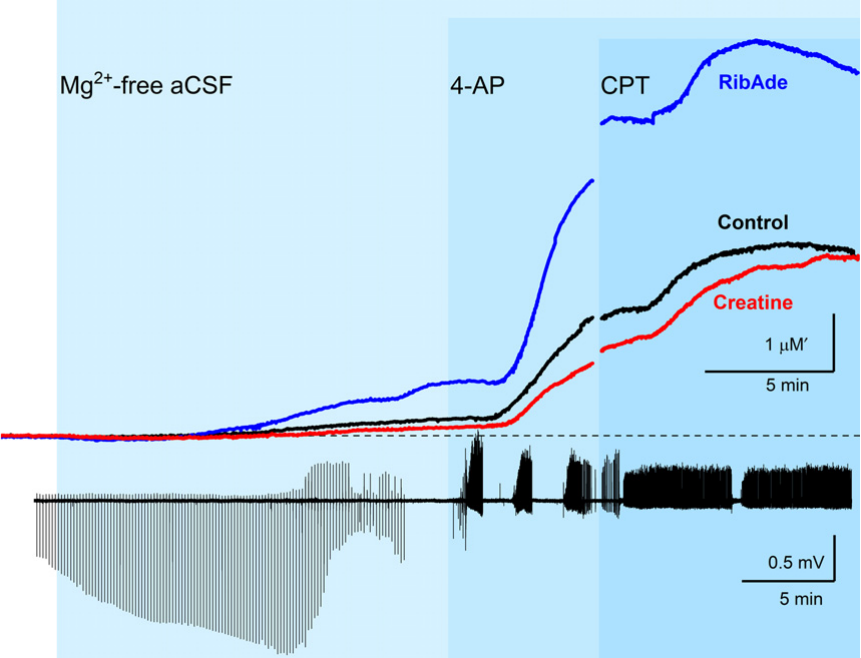
RibAde increased, whereas creatine decreased adenosine release during seizure activity, consistent with their ability to elevate the cellular ATP pool and buffer ATP decline respectively. Upper traces show adenosine release in Mg^2+^‐free artificial cerebrospinal fluid (aCSF), the K^+^ channel blocker 4‐AP (50 μM), and the adenosine A_1_ receptor antagonist 8‐CPT (1 μM) in control (black trace; *n* = 11) and creatine‐ (red trace; *n* = 8) and RibAde‐treated slices (blue trace; *n* = 13). Traces show the averages of between 8 and 13 experiments. The break in the graph between 4‐AP and 8‐CPT reflects the time between the end of either three 4‐AP‐induced bursting episodes or 10 mins in 4‐AP, and the start of 8‐CPT application. The time in 4‐AP thus varied across slices and necessitated synchronization to the time of 8‐CPT application. The lower AC‐coupled electrophysiological trace shows representative synaptic and epileptiform activity associated with the perfusion of slices with Mg^2+^‐free aCSF, 4‐AP and 8‐CPT. Mg^2+^‐free aCSF causes an enhancement of the field excitatory post‐synaptic potential (fEPSP) (Fig. 2d; periodic downward deflections on the trace), which is occasionally curtailed by the rise in extracellular adenosine and the resulting inhibition of the fEPSP (as in this case; see also Lopatar *et al*. 2011). After 15 min in Mg^2+^‐free aCSF electrical stimulation was stopped and 4‐AP was then perfused in the continued absence of extracellular Mg^2+^. 4‐AP‐induced bursting activity interrupted by periods of electrical quiescence (the inter‐burst interval). After either three bursts in 4‐AP or 10 min, 8‐CPT was perfused (in Mg^2+^‐free and 4‐AP‐containing aCSF). 8‐CPT converted the discrete bursting in 4‐AP to sustained firing indicating the high inhibitory adenosine tone in slices.

**Figure 4:**
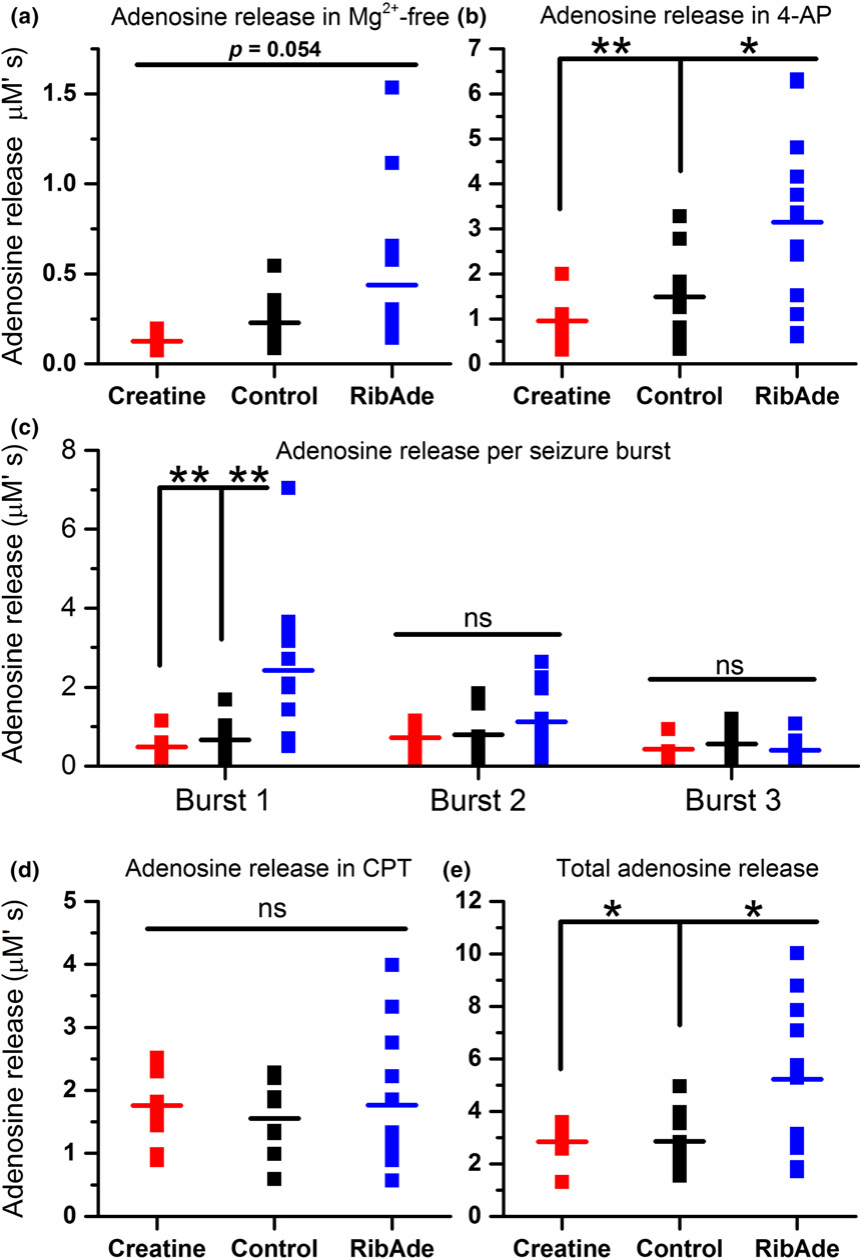
Quantification of adenosine release in control and creatine‐ and RibAde‐treated slices. Area under the curve measurements were made: (a) over 15 min for the adenosine release during initial washout of Mg^2+^ from the slice (*n* = 8–13 slices); (b) after either three bursts or 10 min in 4‐AP (*n* = 8–13 slices); (c) in response to each seizure burst (*n* = 8–13 slices); (d) during challenge with 8‐CPT (*n* = 8–12 slices), (e) over the total adenosine release under each of these conditions (*n* = 8–13 slices). Data are shown from individual experiments, with the mean for each condition and treatment given as the horizontal line. (a) There was no overall group difference in adenosine release in Mg^2+^‐free artificial cerebrospinal fluid (aCSF) (*p* = 0.054), but with a trend towards RibAde‐treated slices releasing most adenosine, and creatine‐treated slices releasing the least. (b) Significant differences in adenosine release were observed in the presence of 4‐AP, in RibAde‐treated slices compared to both control (*p* = 0.018; *) and creatine‐treated slices (*p* = 0.004; **). (c) Individual burst‐induced adenosine release was greatest during burst 1 in RibAde‐treated slices compared to both control (*p* = 0.005; **) and creatine‐treated slices (*p* = 0.005; **). Subsequent bursts 2 and 3 showed no significant differences (ns) in release between control slices and slices pre‐treated with either RibAde or creatine. (d) Although 8‐CPT further increased the amount of adenosine released from slices, this additional release was not significantly different (ns) between treatments (*p* = 0.846). (e) The total combined release per slice was significantly greater in RibAde‐treated slices compared to creatine‐treated slices (*p* = 0.025; *) and control slices (*p* = 0.013; *).

Perfusion of hippocampal slices with Mg^2+^‐free aCSF provoked adenosine release (Lopatar *et al*. 2011) (Fig. 3). The adenosine release in Mg^2+^‐free aCSF was not significantly different across the three groups (Fig. 4a; one‐way anova,* F*_2,29_ = 3.238; *p* = 0.054), but with a trend towards greater release in RibAde‐treated slices (0.44 ± 0.12 μM′s, *n* = 13) and least in slices pre‐incubated with creatine (0.13 ± 0.02 μM′s, *n* = 8). Control slices showed intermediate release of adenosine (0.23 ± 0.04 μM′s, *n* = 11).

Since Mg^2+^‐free aCSF did not consistently result in electrographic seizure activity, we added 4‐AP (50 μM), which provokes epileptiform activity by blocking K^+^ channels and promoting neuronal depolarization (Avoli and Jefferys 2016). 4‐AP caused the appearance of bursting seizure activity and additional adenosine release (Figs 3 and 4b). This release was significantly different across the three groups (one‐way anova,* F*_2,29_ = 7.701; *p* = 0.002), and in RibAde‐treated slices (3.15 ± 0.53 μM′s, *n* = 13) compared to both control (1.49 ± 0.28 μM′s; one‐way anova with post hoc Bonferroni test, *p* = 0.018; *n* = 11) and creatine‐treated slices (0.95 ± 0.18 μM′s; one‐way anova with *post hoc* Bonferroni test, *p* = 0.004, *n* = 8).

Since each burst of seizure activity provoked the release of adenosine, we measured the release of adenosine during each of the three bursts (Fig. 4c). That the release of adenosine occurred in an activity‐dependent manner was evidenced by the fact that there was a linear correlation between seizure duration and adenosine release (*r* = 0.406, *p* = 0.0002, *n* = 82 bursts; data not shown). An analysis of release within each burst showed that while the release of adenosine during bursts 1, 2 and 3 were similar and consistent within creatine‐treated and control slices, RibAde selectively and significantly (one‐way anova:* F*_2,29_ = 8.527; *p* = 0.001) enhanced adenosine release during the first of the three bursts (2.43 ± 0.51 μM′s, *n* = 13) compared to control (0.66 ± 0.18 μM′s; *n* = 11; post hoc Bonferroni: *p* = 0.005) and creatine‐treated slices (0.48 ± 0.11 μM′s, *n* = 8; post hoc Bonferroni test: *p* = 0.005). The fact that greatest release occured only during the first seizure burst in RibAde‐treated slices suggests that there is a rapidly depleting pool of releasable adenosine (Pearson *et al*. 2001; Dale and Frenguelli 2009). Attempts to address this by the continuous perfusion of ribose and adenine during the recording (as opposed to only during pre‐incubation) did not alter the profile of burst‐induced adenosine release, with more adenosine being released only during the first burst (2.54 ± 0.65 μM′s; n = 8; data not shown), suggesting that the interval between bursts (~1–2 mins) was not sufficient to allow replenishment of the ATP‐derived releasable adenosine pool.

The adenosine A_1_ receptor antagonist 8‐CPT (1 μM) was added after slices had displayed three periods of bursting seizure activity, or after 10 mins had elapsed in 4‐AP (Fig. 3) to remove the inhibitory tone caused by ambient adenosine and to thus encourage further epileptiform activity. The addition of 8‐CPT provoked a conversion of bursting seizure activity to continuous spike firing, as described previously (Lopatar *et al*. 2015), and further adenosine release, the extent of which did not differ among the three groups (Fig. 4d; *F*_2,25_ = 0.168, *p* = 0.846; creatine = 1.76 ± 0.23 μM′s, *n* = 8; control = 1.55 ± 0.21 μM′s, *n* = 8; RibAde = 1.77 ± 0.31 μM′s, *n* = 12).

Overall (the sum of release in Mg^2+^‐free, 4‐AP and 8‐CPT conditions; Fig. 4e), RibAde‐treated slices released more adenosine (5.22 ± 0.75 μM′s, *n* = 13; one‐way ANOVAs, *F*_2,29_ = 6.19, *p* = 0.006) than control (2.85 ± 0.32 μM′s, *n* = 11; Bonferroni *post hoc* test, *p* = 0.013) and creatine‐treated slices (2.84 ± 0.26 μM′s, *n* = 8; Bonferroni *post hoc* test, *p* = 0.025). This analysis also held true if only those slices exposed to all three experimental manipulations (Mg^2+^‐free, 4‐AP and 8‐CPT) were considered (one‐way ANOVAs, *F*_2,25_ = 5.818, *p* = 0.008; data not shown).

Slices in which RibAde was continuously perfused throughout this period released even more adenosine (8.33 ± 1.17 μM′s; *n* = 8; not shown). This latter observation suggests that, over the time‐course of these experiments (~30 mins), the replenishment of an adenine nucleotide pool that contributes to activity‐dependent adenosine release can occur. This has potential implications for conditions, such as TBI, where post‐traumatic seizures can persist for prolonged periods and may initiate epileptogenesis.

### Creatine and RibAde influence seizure activity by regulating the availability of extracellular adenosine

**Figure 5:**
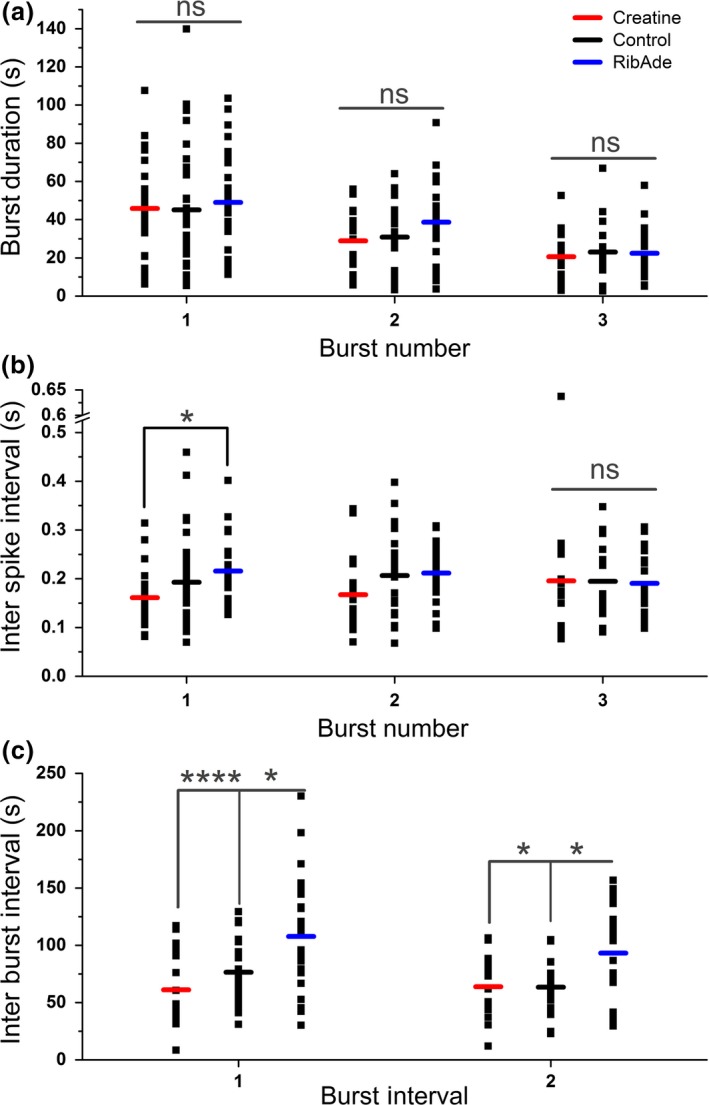
Seizure frequency and intensity, but not duration, is influenced by pre‐incubation with creatine and RibAde. (a) The duration of 4‐AP‐induced bursts was not significantly different (ns) between the three treatments (Burst 1: *p* = 0.848, *n* = 24–32 slices; Burst 2: *p* = 0.150, *n* = 19–27 slices; Burst 3: *p* = 0.883, *n* = 15–22 slices). (b) Treatment influenced the inter‐spike interval in Burst 1 (*p* = 0.028), with a significant difference between RibAde‐ and creatine‐treated slices (*p* = 0.023; *), but with no influence on Bursts 2 or 3. (c) Inter‐burst interval was sensitive to the treatments. RibAde delayed the occurrence of Burst 2 (Inter‐Burst Interval 1; *n* = 19–27) compared to both creatine‐treated (*p* < 0.0001; ****) and control slices (*p* = 0.018; *). Burst 3 (Inter‐Burst Interval 2; *n* = 15–22) was also delayed in RibAde‐treated slices compared to both creatine‐treated (*p* = 0.031; *) and control slices (*p* = 0.019; *).

To establish whether the modulation of adenosine release by creatine and RibAde influenced seizure activity we measured a number of parameters of the bursting activity evoked by Mg^2+^/4‐AP: duration of seizure bursts (Fig. 5a), the inter‐spike interval during a burst (Fig. 5b), and the time between bursts, the inter‐burst interval (Fig. 5c).

Neither creatine nor RibAde influenced the duration of the first, second or third seizure bursts (Fig. 5a; one‐way ANOVAs, *F*_2,83_ = 0.166, *p* = 0.848; *F*_2,65_ = 1.954, *p* = 0.150; *F*_2,51_ = 0.124, *p* = 0.883 respectively). Within each of the three bursts, RibAde increased the inter‐spike interval (i.e. reduced burst intensity) compared to creatine only during the first burst, where greatest release of adenosine was observed (Fig. 4c, 5b; one‐way anova,* F*_2,83_ = 3.728, *p* = 0.028, post hoc Bonferroni RibAde vs. creatine, *p* = 0.023). To test whether creatine and RibAde had an effect on the frequency at which bursting occurred, the inter‐burst interval was measured (Fig. 5c). The time of occurrence of the second burst (Inter‐Burst Interval 1) in RibAde slices was delayed (107.8 ± 9.3 s) compared to both creatine‐treated (61.2 ± 7.4 s; *Post hoc* Bonferroni: *p* < 0.0001) and control slices (76.5 ± 5.8 s; *post hoc* Bonferroni: *p* = 0.018; one‐way anova:* p* < 0.0001; *F*_2,65_ = 8.983). Similarly, the time to the third burst (Inter‐Burst Interval 2) was increased (one‐way anova:* p* = 0.008; *F*_2,50_ = 5.398) in RibAde‐treated slices (93.2 ± 8.4 s) compared to creatine (63.9 ± 7.6 s; post hoc Bonferroni: *p* = 0.031) and control slices (63.5 ± 5.8 s; post hoc Bonferroni: *p* = 0.019).

## Discussion

We have shown that the accumulation of extracellular adenosine during brief seizure activity is dependent upon the availability and metabolism of intracellular ATP. This observation in itself suggests that synapses are under autonomous, spatially restricted, activity‐dependent control that is determined by local cellular ATP levels and its rapid metabolism to adenosine. Moreover, we show that the availability of ATP can be modulated pharmacologically. This may be of value in conditions characterized by ATP depletion where ATP levels, and the reservoir for the anticonvulsant adenosine, may be restored via the provision of ribose and adenine.

## Restoration of cellular ATP via the purine salvage pathway

The brain, like the heart, relies upon the purine salvage pathway for the synthesis of adenine nucleotides (Ipata *et al*. 2011; Frenguelli 2017). This synthesis initially occurs in the form of AMP, either through the actions of adenine phosphoribosyl‐transferase and HPRT on nucleotide precursor molecules, or via the action of adenosine kinase (EC 2.7.1.20) on adenosine (Fig. 1). While this route is rapid, energetically efficient, occurs in the cytosol and can deliver ATP to remote neuronal regions lacking mitochondria such as dendritic spines; (Kasthuri *et al*. 2015; Babits *et al*. 2016), it is constrained by the availability of salvageable substrates. Under normal circumstances this constraint is met, and substrates are available in sufficient quantities to meet the demand for ATP. However, under conditions of ATP depletion, most notably cerebral ischaemia, ATP is metabolized to compounds that are lost to the circulation, or, in the case of xanthine, beyond salvage (Weigand *et al*. 1999; Frenguelli 2017; Tian *et al*. 2017). This loss of salvageable substrates, together with injury‐induced mitochondrial dysfunction, and indeed potential ATP *consumption* by mitochondria, likely explains the profound and protracted depletion of cerebral ATP after various forms of injury (Frenguelli 2017).

Using hippocampal slices as a model of the post‐traumatic, post‐ischaemic brain (Hossmann 2008) we previously showed that providing slices with the ATP precursors ribose and adenine (RibAde) resulted in an elevation of tissue ATP to levels found *in vivo* (zur Nedden *et al*. 2011; zur Nedden*et al*. 2014). Moreover, this elevation of cellular ATP increased the reservoir for adenosine, which was released in greater quantities during high‐frequency stimulation of afferents (zur Nedden *et al*. 2011) and during oxygen/glucose deprivation (OGD) (zur Nedden*et al*. 2014). Creatine, on the other hand, did not elevate tissue ATP, but did preserve cellular ATP during OGD and, as a consequence, reduced adenosine release (zur Nedden*et al*. 2014). In this study, using the concentrations and duration of application of RibAde and creatine described previously, we confirmed the bidirectional modulation of adenosine release by RibAde and creatine, which we have previously shown by HPLC was because of elevation and preservation, respectively, of cellular ATP (zur Nedden *et al*. 2011; zur Nedden*et al*. 2014). We have also confirmed that these manipulations do not affect basal synaptic transmission, paired‐pulse facilitation or the pre‐synaptic fibre volley; their influence is therefore only likely to be manifest under conditions of ATP depletion, in the present case during epileptiform activity.

## Seizure activity‐dependent adenosine release

Removal of extracellular Mg^2+^ favours both activation of post‐synaptic NMDA receptors and promotes glutamate release. As such it is commonly used as a means to increase the excitability of neuronal tissue (Mody *et al*. 1987). This excitation is tempered by the NMDA receptor‐dependent release of adenosine (Manzoni *et al*. 1994), which we have previously been able to observe directly using adenosine biosensors (Lopatar *et al*. 2011). In this study, there was a tendency for the release of adenosine in Mg^2+^‐free aCSF to be bidirectionally influenced by both RibAde and creatine, with greater release of adenosine in RibAde‐treated slices, and less release from slices pre‐incubated in creatine. These observations are consistent with the ability of these compounds to elevate and buffer cellular ATP, respectively.

Inclusion of the K^+^ channel blocker 4‐AP in the Mg^2+^‐free aCSF resulted in the appearance of bursting epileptiform activity (Avoli and Jefferys 2016). This manipulation provoked further adenosine release, which was similarly influenced by creatine (reduced) and RibAde (increased). An analysis of adenosine release on a burst‐by‐burst basis showed that, while consistent release was observed across the three bursts in creatine‐treated and control slices, RibAde‐treated slices released substantially more adenosine on the first burst, before releasing similar levels of adenosine during the subsequent two bursts. This observation is consistent with the concept of a depletable pool of adenosine that we have described previously (Pearson *et al*. 2001) and implies that there is a readily releasable pool of adenosine, most likely in the form of intracellular ATP, which, once released, takes some time to be replenished. Indeed, attempts to replenish this pool between bursts with continuous presentation of RibAde did not affect the profile of burst‐induced adenosine release, suggesting that the 1–2 min inter‐burst interval is not sufficient time for replenishment to occur, even in the presence of substrate. Previous successful attempts to replenish the adenosine pool have involved prolonged intervals between challenges, the provision of exogenous adenosine, and β adrenoceptor activation (Pearson *et al*. 2001; Pearson and Frenguelli 2004; Dale and Frenguelli 2009). The ability to restore adenosine release suggests that adenosine depletion does not reflect an irreversible or pathological condition, but more likely a diminution of locally available ATP or its precursors. Indeed, in this study, slices continuously perfused with RibAde released more adenosine overall than did slices that had only been pre‐incubated in RibAde, suggesting that over the time‐course of these experiments the releasable pool of adenosine can be replenished.

The adenosine A_1_ receptor antagonist 8‐CPT converted 4‐AP‐induced bursting activity into regular spiking. This implies that the duration and timing of bursts is regulated by the burst‐by‐burst release of adenosine and the subsequent activation of A_1_ receptors. Such conversions from bursting to regular spiking have been observed previously *in vitro* (Avsar and Empson 2004; Lopatar *et al*. 2015) and have been described as akin to entry into a status epilepticus‐like state (Avsar and Empson 2004). *In vivo*, knockout of adenosine A_1_ receptors leads to fatal status epilepticus after both intra‐hippocampal kainic acid (Fedele *et al*. 2006) and traumatic brain injury (Kochanek *et al*. 2006). In humans, the adenosine receptor antagonist theophylline can precipitate status epilepticus (Kohl *et al*. 2011). These observations indicate the importance of adenosine A_1_ receptors in limiting, localizing and terminating seizure activity.

The increased spiking caused by 8‐CPT resulted in greater adenosine release, but the quantities released did not differ across the three treatment groups. It is somewhat surprising that more adenosine was not released in RibAde‐treated slices during 8‐CPT since there was more adenosine release in these slices during both Mg^2+^‐free and Mg^2+^‐free/4‐AP perfusion. It is possible that the larger quantities of adenosine released previously depleted the releasable pool of adenosine described above. Nonetheless, the total quantity of adenosine release was greater in RibAde‐treated slices, and yet more was released from slices continuously perfused with RibAde. This latter observation does suggest that there may be value in providing the injured brain with the substrates to make ATP and thus to boost the cellular reservoir of the endogenous anticonvulsant adenosine.

## Modulation of intracellular ATP influences epileptiform activity

We have previously shown that pharmacological modulation of intracellular ATP influences adenosine release, and through this both synaptic transmission and synaptic plasticity. Pre‐treatment of hippocampal slices with RibAde raised the threshold for the induction of long‐term potentiation in a manner that was dependent upon the activation of adenosine A_1_ receptors (zur Nedden *et al*. 2011), whereas the depressant effects of OGD on hippocampal synaptic transmission, which are known to be largely dependent upon adenosine A_1_ receptors both *in vitro* and *in vivo* (Fowler 1990; Gervitz *et al*. 2001; Dale and Frenguelli 2009), were enhanced by RibAde (zur Nedden*et al*. 2014). In contrast, as has been observed by others (Whittingham and Lipton 1981; Okada and Yoneda 1983), creatine delayed the depression of synaptic transmission by OGD (zur Nedden*et al*. 2014). This is consistent with the better preservation of cellular ATP and the reduced efflux of adenosine, rather than, as has been proposed, better maintenance of cellular ATP per se. The preservation of cellular ATP by creatine did, however, delay the appearance of the anoxic depolarization and allowed the recovery of synaptic activity after prolonged OGD (zur Nedden*et al*. 2014), confirming previous *in vivo* and *in vitro* observations of the benefits of creatine (Krivanek *et al*. 1958; Whittingham and Lipton 1981; Okada and Yoneda 1983; Balestrino 1995; Balestrino *et al*. 1999).

In this study we considered the actions of creatine and RibAde on electrographic epileptiform activity. We measured three parameters relating to 4‐AP‐induced seizure activity: burst duration; inter‐spike interval and inter‐burst interval. We found that neither creatine nor RibAde influenced the duration of the three bursts evoked by 4‐AP. This is somewhat surprising given the differential effects of these manipulations on adenosine release, and the importance of adenosine A_1_ receptors in terminating the bursts (see above). One possible explanation is that even under conditions of restricted ATP metabolism (by creatine) sufficient adenosine is released to terminate seizures; any additional release in RibAde‐treated slices is supramaximal and thus has no additional benefit in seizure termination. However, this is not to say that this additional release is without effect; a comparison of the inter‐spike intervals within each burst showed that RibAde slowed the frequency with which spikes occurred within the first burst – the burst during which greatest adenosine release was observed in RibAde‐treated slices. Thus, while the duration of the burst is unaffected, the intensity of the burst is reduced. Moreover, the interval between the bursts was increased by RibAde, thereby reducing the frequency with which bursts appear. This is consistent with the greater accumulation of adenosine caused by a burst taking longer to return to a level below the threshold for subsequent burst initiation.

## Therapeutic implications of pharmacological modulation of cellular ATP

There has been considerable interest in creatine as a neuroprotective agent. This has arisen through a number of *in vitro* and preclinical studies showing: creatine‐mediated restoration of cerebral phosphocreatine (Thomas 1956; Whittingham and Lipton 1981; Yoneda *et al*. 1983); preservation of neuronal membrane potential in the face of metabolic stress (Krivanek *et al*. 1958; zur Nedden*et al*. 2014) recovery of synaptic function after OGD (Whittingham and Lipton 1981; Okada and Yoneda 1983; Balestrino 1995; Balestrino *et al*. 1999; zur Nedden*et al*. 2014), and neuroprotective benefits of creatine following pre‐treatment *in vivo* (Andres *et al*. 2008; Balestrino *et al*. 2016). Pre‐treatment is necessary given the lack of creatine transporters on astrocytic endfeet, which surround cerebral blood vessels and thus hamper ingress of creatine (Andres *et al*. 2008; Balestrino *et al*. 2016). This limitation in the potential of creatine as a therapy has led to both the development of more cell‐permeable derivatives of creatine and calls for prophylactic therapy for individuals at high risk of brain injury, for example as a consequence of stroke (Balestrino *et al*. 2016).

Alternatively, we have proposed the use of ribose and adenine, either alone or in combination with a xanthine oxidase inhibitor, to improve outcome after brain injury (Frenguelli 2017). In a rodent model of ischaemic stroke, RibAde, when given intravenously for 6 hr during reperfusion, showed an encouraging trend to reduce brain lesion volume and accelerate recovery of function in the week after the period of cerebral ischaemia (Faller *et al*. 2017). Inclusion of a single intra‐peritoneal injection of the xanthine oxidase inhibitor, allopurinol (‘RibAdeAll’), resulted in protection of a larger volume of brain tissue (Faller *et al*. 2017). This enhancement by allopurinol may reflect: reduced production of non‐salvageable xanthine allowing more hypoxanthine for salvage and ATP synthesis; reduction in the production of reactive oxygen species derived from hydrogen peroxide, or a direct anti‐oxidant action of allopurinol or its active metabolite oxypurinol (Juul and Ferriero 2014; Frenguelli 2017).

Given that: (i) ribose has been used in man as an unregulated nutritional supplement and as an aid to cardiac rehabilitation and exercise performance (Bayram *et al*. 2015; Seifert *et al*. 2017); (ii) adenine is added to blood transfusion products as erythrocytes lack mitochondria and synthesize ATP via purine salvage, and (iii) allopurinol has been in use for the treatment of gout since the 1960s, suggests that the combination of these compounds are likely to be well‐tolerated by humans and may form the basis of a readily deliverable treatment for the hyper‐acute management of brain injury.

## Conclusions

We have shown that the combined application of ribose and adenine (RibAde) promoted greater accumulation of extracellular adenosine during electrographic seizures in hippocampal slices. This resulted in a reduction in the intensity and frequency of epileptiform activity. These observations, together with our previous studies *in vitro* and *in vivo* in a rodent stroke model, and their safe use in humans, suggest that ribose and adenine, together with allopurinol, may be of value in the acutely injured brain.

## Acknowledgments and conflict of interest disclosure

JH was supported by a BBSRC‐funded Doctoral Training Program. BGF is a Non‐Executive Director and shareholder of Sarissa Biomedical Ltd, the company that manufactures the microelectrode biosensors used in this study.
